# The Effect of Indigenous Cultivable Microorganism Inoculation on Soil Microecology During Restoration of Obstructed Soils

**DOI:** 10.3390/microorganisms14040784

**Published:** 2026-03-30

**Authors:** Qunfei Ma, Bing Zhang, Juntao Cui

**Affiliations:** College of Resources and Environment, Jilin Agricultural University, Changchun 130118, China

**Keywords:** microbial inoculation, ecological restoration, soil health, environmentally friendly

## Abstract

Soil fumigation effectively mitigates replanting obstacles induced by intensive cultivation, yet its non-targeted biocidal effects can suppress beneficial microbial activity, potentially compromising agricultural sustainability. Microbial inoculation, as a strategy to supplement beneficial microorganisms, is often employed to restore soil microbial communities. However, in practice, commonly used exogenous microbial consortia exhibit poor adaptability in non-native environments, frequently resulting in limited efficacy. To address this limitation, we propose an ecological intervention based on the reintroduction of indigenous cultivable microorganisms: cultivable microbial communities were isolated from healthy adjacent soils and inoculated into fumigated soils affected by replanting obstacles. The experimental soil consisted of black soil under continuous cropping, collected from Northeast China. The three treatments were continuous cropping soil (control), fumigated continuous cropping soil and fumigated continuous cropping soil after inoculation of indigenous cultivable microorganisms. Using high-throughput sequencing and agronomic–chemical analyses, combined with cross-domain networks and procrustes analysis, we systematically assessed the ecological effects of this approach on microbial restoration and the alleviation of replanting obstacles. The results showed that indigenous cultivable microorganism inoculation significantly increased the richness of bacterial and fungal communities in fumigated soils within 21 days, extending microbial richness and diversity. Furthermore, inoculation accelerated the reconstruction of dominant microbial community structures, with the relative abundance of dominant species reaching up to 80%. Positive synergistic interactions between bacteria and fungi increased by approximately 10%, enhancing network stability. Key bacterial taxa, such as *Paenibacillus* and *Mycobacterium*, were significantly correlated with available potassium and phosphorus content, while *Micromonospora*, *Massilia*, and *Flavisolibacter* influenced plant fresh weight, total nitrogen, and potassium accumulation. Key fungal taxa, such as *Cryptococcus* and *Phialemonium*, were significantly associated with soil organic matter stability, maize photosynthetic efficiency, plant dry weight, and total phosphorus content. This study confirms the ecological adaptability and functionality of indigenous cultivable microorganisms in soil ecosystem restoration, offering a low-risk, highly effective localized intervention strategy for sustainable agriculture.

## 1. Introduction

The model of intensive farming is commonly used in agriculture production practices in contemporary time with the maximum use in facility agriculture systems and economic crop farming systems due to the ability to significantly increase the economic benefit of land [1]. However, long-term continuous cropping usually results in continuous cropping obstacles, which are the declining yield of crops [2], increased soil-borne diseases [3] and degrading soil functions [4]. Previous studies indicate that continuous cropping constraints originate primarily from the accumulation of harmful microorganisms, the weakening of nutrient cycling, soil structural degradation that continuously progresses, and the culmination of a state of soil ecosystem imbalance, especially disruption of the soil microbe community and lowering functional diversity [5,6,7,8].

Soil disinfection has emerged as a crucial method for reducing continuous cropping obstacles and removing pathogens [9]. Fumigation technology has become one of the mainstream measures for regulating continuous cropping obstacles because of its ease of operation, fast action, and broad-spectrum inhibition towards various soil-borne pathogens [10]. The untargeted sterilizing effects caused as a result of fumigating non-selectively affect beneficial microbial communities in the soil, which play key roles in carbon and nitrogen cycling, pathogen antagonism, and nutrient supply to plants [11]. Beneficial microorganisms, including nitrogen-fixing bacteria such as *Rhizobium* and *Bradyrhizobium*, phosphorus-solubilizing bacteria like *Bacillus* and *Pseudomonas*, as well as fungal communities from Ascomycota and Basidiomycota, are also affected [12,13,14]. After fumigation, the diversity and metabolic activity of these functional microbial groups decrease significantly, which delays microbial community recovery, weakens soil ecological functions, and affects the long-term productivity of crops [15,16].

The application of microbial inoculation technologies (such as functional inoculants and composite inoculants) is an effective measure for the recovery of microbial functions in fumigated soils [17]. The methods aim to supplement and increase the population of functional microbes in the soil by adding exogenous functional microbes. This enhances the health and fertility of the soil [18]. Angelina and Ng found that further increasing inoculation with exogenous functional microorganisms can induce faster recovery of beneficial microbes such as decomposition microbes, nitrogen-fixation microbes, and phosphorus-solubilizing microbes, improving soil nutrient transformation and pathogen antagonism and upgrading the functionality of a particular ecosystem [19,20]. It must be noted that most of these exogenous inoculants consist of commercial strains or artificially selected microbial communities. The presence of a vast overlap of ecological niches and competition with native microorganisms for resources often leads to poor colonization stability in the soil by these inoculants, limited expression of functions and even disturbance of the native microbiota [21,22]. Research by Thakur suggests that even if exogenous inoculants performed well in the lab, their performance in the fields is generally poor due to lack of adaptability. This limits their sustainable usage for resolving continuous cropping issues [23].

As a result, there is an urgent requirement to formulate soil microbial restoration strategies that target greater ecological adaptability and system stability for efficient reconstruction of soil ecological functions under continuous cropping conditions [24,25]. In light of the aforementioned challenges, the study proposes a microecological intervention strategy “the reintroduction of healthy indigenous cultivable microorganisms”. This strategy is mainly based on isolating and selecting indigenous cultivable microorganisms from healthy soils free from continuous cropping obstacles in the same ecological zone that will be inoculated into fumigated soils that were previously afflicted with continuous cropping problems. The goal is to promote the rapid reconstruction of community structures and regeneration of core functions of microbes [26]. This approach emphasizes the flexibility of in situ management and the importance of ecological niche matching, relying on local microbial resources to minimize the potential ecological risks associated with exogenous inoculants [27]. Based on this principle, we hypothesize that introducing indigenous cultivable microorganisms can accelerate the reconstruction of soil microbial communities, effectively enhance soil functionality, and strengthen sustainable productivity. This study aims to systematically evaluate the potential of this strategy to restore microbial communities and alleviate soil constraints, providing a scientific basis for the development of low-risk, environmentally friendly soil management practices while highlighting its practical significance for ecological intervention and soil health restoration.

## 2. Materials and Methods

### 2.1. Sampling Site Description

Soils collected from fields with continuous cropping problems at Jilin Agricultural University and adjacent healthy uncultivated soils were used for the cultivation experiment. In the spring of 2024, both soils were sampled at random in an S-shape. For each soil, five sampling points were chosen, and the sample was taken at three different depths, namely 0–20 cm, 20–40 cm and 40–60 cm. Samples from different sampling points and depths were thoroughly mixed to represent the entire soil system. Plant debris and crushed stones were removed, and the samples were taken back to the laboratory. These samples were utilized to simulate full-profile soil conditions. The specific sampling procedure was performed as shown in Figure S1. The sampling site sampling region was in Changchun, China (43°48′43.57″ N, 125°23′38.50″ E, Figure S2), where the climate belongs to the temperate continental monsoon climate zone. The total average annual rainfall is 400–620 mm, with 60% of the rainfall during summer. The frost-free period varies from 135–155 days, with an average daily duration of sunlight of 7 h. The yearly temperature usually lies in the range of 3–5 °C. The minimum and maximum air temperatures in the experimental site reached −30 °C and 33 °C, respectively. The soil type was classified as black soil, corresponding to Argiudolls in the United States soil taxonomy. The basic physicochemical properties of the soil were as follows: pH 6.5, organic matter (OM) 22.55 g kg^−1^, alkaline hydrolyzable nitrogen (AN) 110.11 mg kg^−1^, available phosphorus (AP) 51.72 mg kg^−1^, and available potassium (AK) 146.05 mg kg^−1^.

### 2.2. Experimental Design

The study involved three treatments, which were (1) continuous cropping soil (CCS), (2) fumigated continuous cropping soil (FUM) and (3) fumigated continuous cropping soil after inoculation of indigenous cultivable microorganisms from uncultivated healthy soil (FUM + MIC). Each of the treatments was replicated 9 times. Each replicate was placed in a sterile cultivation container of the same size. In all containers, the soil corresponding to the treatments was taken in equal weight and incubated for 7 days to stabilize the microenvironment, after which the soils were used for the cultivation of radish (Raphanus sativus). The study took place in a greenhouse with regulated temperature, maintaining 25 °C and featuring a light cycle consisting of 8 h of light followed by 16 h of darkness.

Sterilization of the FUM treatment was done by the chloroform vapor fumigation method. The microorganisms used in the FUM + MIC treatment were indigenous cultivable microorganisms. Indigenous cultivable microorganisms from uncultivated healthy soil were isolated through the conventional dilution plate method and cultured on three different media, i.e., beef extract peptone agar, Czapek’s agar, and modified Gao I agar. The culture medium composition was prepared as detailed in Table S1. After 2 to 15 days of incubation in an inverted position, microbial colony counts were performed to determine the ratios of bacteria, fungi and actinomycetes. These microbial groups were washed with sterile water, mixed and shaken for a period of 30 min. After removing the supernatant by centrifugation at 1500 rpm for 2 min, microorganisms were added back into liquid media according to the treatment and incubated at 28 °C on a shaker for 7 days. Liquid beef extract peptone agar, liquid Czapek’s agar, and liquid modified Gao I agar were used to culture bacteria, fungi, and actinomycetes, respectively. Before utilization, the microbial suspension was centrifuged at 10,000 rpm for removal of contaminants and washed twice with enzyme-free sterile water. The suspension was then restored to the proportions of the original isolation and inoculated into the fumigated soil.

### 2.3. Sample Collection and Preparation

Soil samples were collected on day 0, day 21 and day 90. The samples were split into two parts after sampling. One fraction was air dried, ground and sieved with a 2 mm sieve for analysis of soil physical–chemical properties. The leftover portion was stored at −80 °C for later extraction of soil DNA and for further analysis of its microbial community composition.

Samples from the plants were taken on day 90. The relative chlorophyll content (SPAD) of the plant leaves was measured before harvesting. After harvesting, both the aerial and subterranean parts of the plants were weighed in a fresh and dry state for biomass assessment. Following the drying process, the samples were sifted to assess the levels of total nitrogen (TN), total phosphorus (TP), and total potassium (TK).

### 2.4. Sample Analysis

Soil organic matter (OM) was determined using the K_2_Cr_2_O_7_ oxidation method [28]. Alkali-hydrolysable nitrogen (AN) was measured using the alkali diffusion method (Nelson and Sommers, 1982) [29]. Available phosphorus (AP) was extracted using NaHCO_3_ and quantified according to the method of Olsen et al. (1954) [30]. Available potassium (AK) was extracted using 1 mol·L^−1^ NH_4_Ac, and the potassium content was determined after filtration of the extract using a flame photometer (Hitachi Z-2000, Tokyo, Japan) [31].

The SPAD value of plant leaves was measured using a portable SPAD-502 chlorophyll meter (Konica Minolta, Tokyo, Japan) [32]. After harvesting, the fresh weight (FW) and dry weight (DW) of the aboveground and underground parts of the plants were measured to assess plant biomass [33]. The TN content in the plants was determined using the Kjeldahl method [34], TP was measured by molybdenum–antimony colorimetry following sulfuric acid–perchloric acid digestion [35], and TK was quantified using flame photometry [36].

### 2.5. Soil Total DNA Extraction, Sequencing and Analysis

The total DNA was extracted from fresh soil samples of microorganisms using a MagBeads FastDNA Kit for Soil (MP Biomedicals, Irvine, CA, USA), according to the guidelines provided by the manufacturer. The integrity of the isolated DNA was evaluated through 0.8% agarose gel electrophoresis, while its concentration and purity were assessed using a Nanodrop UV spectrophotometer (Thermo Scientific, Waltham, MA, USA).

To analyze the bacterial community, amplification was carried out on the V3-V4 hypervariable region of the 16S rRNA gene, which is about 480 bp in length. PCR amplification was performed using the primers 338F (5′-barcode-ACTCCTACGGGAGGCAGCA-3′) and 806R (5′-GGACTACHVGGGTWTCTAAT-3′). For fungal community analysis, the ITS gene V1 region (approximately 280–500 bp) was targeted, with amplification performed using the primers ITS5 (5′-GGAAGTAAAAGTCGTAACAAGG-3′) and ITS2 (5′-GCTGCGTTCTTCATCGATGC-3′). Library construction and Illumina high-throughput sequencing were carried out by Shanghai Paigen Biosciences Co., Ltd. (Shanghai, China).

The QIIME2 platform (v 2022-11) was utilized for the processing of raw sequencing data. The initial step was the demultiplexing of sequences using the demux plugin. Next, the cutadapt plugin removed primer sequences, and unmatched reads were filtered out. Utilizing the DADA2 plugin, we performed quality assessment, denoising, merging of paired ends, and chimera elimination to produce amplicon sequence variants (ASVs) along with abundance tables. Upon independent processing of the samples, the ASV tables were merged, and singleton ASVs were discarded. Using R (v 4.3.0), we performed sequence length distribution statistics. To further optimize data quality, secondary quality control was performed using VSEARCH. Paired-end merging was performed using the fastq_mergepairs module, quality filtering was done using the fastq_filter module, and duplicate sequences were removed using the derep_fulllength module. Preliminary clustering was performed at 98% similarity using the cluster_size module, followed by chimera removal using the uchime_denovo algorithm. Finally, the remaining high-quality sequences were clustered at 97% similarity, and representative sequences and corresponding ASV abundance tables were output.

The RDP Classifier (v 2.2) was utilized for the taxonomic classification process. The alignment of bacterial 16S rRNA sequences was conducted with the Greengenes database (Release 13.8), while the fungal ITS sequences were arranged according to the UNITE database (Release 8.0) [37,38].

### 2.6. Statistical Analysis

The evaluation of statistical significance among various treatments was conducted through analysis of variance (ANOVA) along with multiple comparisons, specifically using Duncan’s new multiple range test, at a significance level of *p* < 0.05 (*, *p* < 0.05; **, *p* < 0.01; ***, *p* < 0.001). All statistical analyses were carried out utilizing IBM SPSS Statistics software (v 25.0) [39]. Soil nutrient contents (OM, AN, AP, and AK), plant nutrients and traits (DW, FW, SPAD, TN, TP, and TK), microbial alpha diversity indices (Chao1 and Shannon) were visualized as boxplots using the ggplot2 package in R (v 4.3.3). The species accumulation bar plots for microbial communities were also generated. Beta diversity analysis of microbial communities was based on Bray–Curtis dissimilarity matrices, with principal coordinate analysis (PCA) performed using the vegan package in R (v4.3.3), and the statistical significance of community structure differences between treatments was tested using ANOSIM.

The WGCNA package in R (v 4.3.3) was utilized to establish networks of microbial co-occurrence. The topological properties of the overall network and its subnetworks for different treatments were quantified and evaluated using the igraph package. Metrics for network analysis comprised the count of nodes and edges, as well as the average density, average path length, and the diameter of the network [40]. Network structure was visualized using Gephi software (v 0.9.2). To identify microbial groups with key ecological functions in the network, further analysis was performed using the R script zipi.r to calculate the Zi (within-module connectivity) and Pi (among-module connectivity) values of the network nodes. The Zi–Pi analysis method, as proposed by Guimerà and Nunes, was employed to classify nodes within the microbial network. Zi represents the degree of connectivity of a node within its module, while Pi indicates the degree of connectivity between different modules. Based on the combinations of Zi and Pi values, nodes were classified into 4 ecological roles: (1) module hubs, key nodes with high connectivity within the module (Zi > 2.5 and Pi < 0.62); (2) connectors, bridge nodes with significant connectivity between modules (Zi < 2.5 and Pi > 0.62); (3) network hubs, global central nodes with high connectivity both within and between modules (Zi > 2.5 and Pi > 0.62); (4) peripherals, edge nodes with low connectivity both within and between modules (Zi < 2.5 and Pi < 0.62) [41]. Within the Zi–Pi framework, module hubs serve as stabilizers of local networks, dominating the ecological processes within their modules. Connectors ensure the flow of information and resources across modules by bridging them. Network hubs are central nodes that maintain the stability and functionality of the ecosystem, preserving the connectivity of the network. Therefore, species in module hubs, connectors, and network hubs are considered keystone species, playing vital roles in maintaining network structure, facilitating resource flow, and ensuring system stability [42]. Visualization was performed using the “ggplot2” package.

Finally, the network node information was integrated with microbial species annotation data to identify keystone species in different modules. Cross-domain network analysis of fungal and bacterial keystones in different treatments was performed based on the phyloseq, igraph, network, sna, tidyverse, ggClusterNet, dplyr, and ggplot2 packages in R (v 4.3.3) [43]. The regional IDEN threshold was set to 0.80, with significance at 0.05, to filter out irrelevant associations. The adjacency matrix obtained from the bipartite graph consisted of 1s or 0s, indicating the presence or absence of associations between fungal keystone and bacterial keystone species. Visualization was carried out using the Cytoscape software (v 3.7.1).

A procrustes analysis was conducted with the procrustes function from the vegan package in R (v 4.3.3) to investigate the relationship between shifts in the abundance of microbial keystones and variations in soil nutrient content, along with the nutrient and trait values of plants [44]. Network heatmaps were constructed using the linkET package in R (v 4.3.3) to determine the specific relationships between microbial keystones and soil nutrient contents as well as plant nutrient and trait changes [45]. Heatmaps illustrating correlations were generated with the heatmap package in R (v 4.3.3) to identify specific genus-level keystones related to soil nutrient content and plant nutrient and trait variations.

## 3. Results

### 3.1. Soil Nutrient Content Dynamics

**Figure 1:**
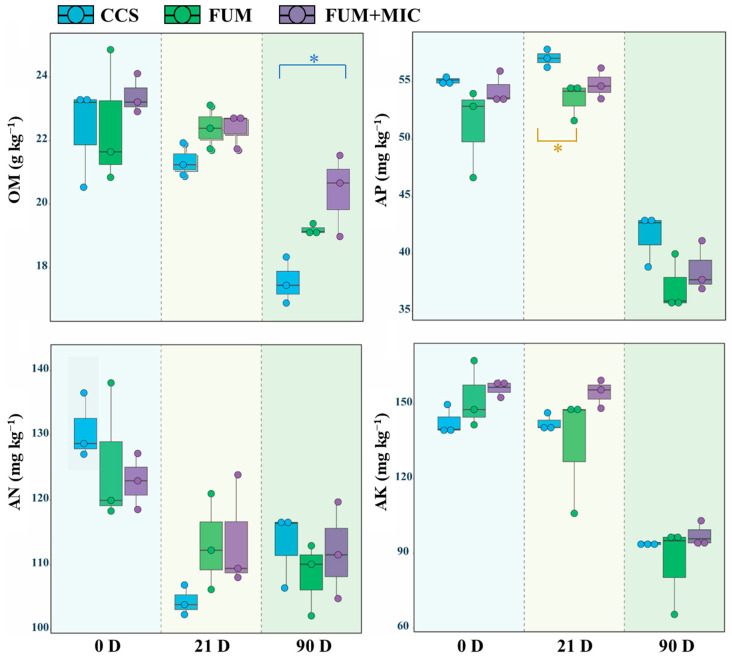
Changes in soil nutrient content during different cultivation periods under various treatments in a model field experiment. Note: asterisks denote significant probability levels.

Different treatments and cultivation durations significantly affected soil nutrient contents (Figure 1). On day 21, compared to the CCS treatment, the FUM treatment significantly reduced the AP content by 6.21% (*p* < 0.05). On day 90, compared to the CCS treatment, the FUM + MIC treatment significantly increased the OM content by 4.66% (*p* < 0.05). The soil nutrient contents in all treatments decreased over time as the cultivation period was extended. The most substantial alteration in AN content occurred on day 21, while a vast majority of changes in OM, AP, and AK contents occurred mainly on day 90.

### 3.2. Characteristics of Plant Growth and Nutritional Content

**Figure 2:**
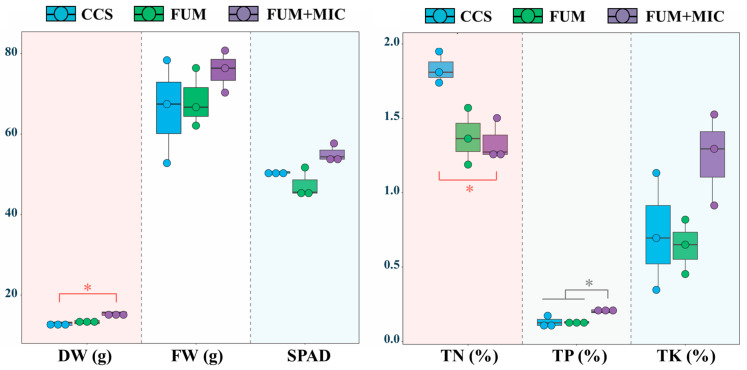
Changes in agronomic traits and nutrient contents of plants on day 90 under various treatments in a model field experiment. Note: asterisks denote significant probability levels.

There were great differences in plant agronomic traits and nutrient accumulation under various treatments (Figure 2). In terms of agronomic characteristics, FUM + MIC treatment showed a very significant increase of 19.45% in the DW content over CCS. In terms of nutrient accumulation, the TN content was significantly reduced by 26.91% in the FUM + MIC treatment compared to CCS. Also, the TP content in the FUM + MIC treatment increased by 59.44% and 61.25% compared to the CCS and FUM treatment, respectively.

### 3.3. Differential Responses of Microbial Alpha Diversity

**Figure 3:**
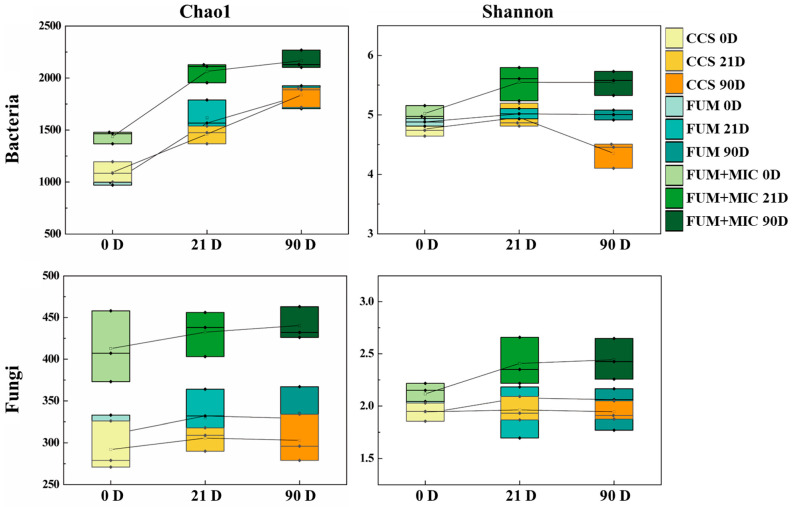
Changes in the alpha diversity of soil microbial communities under different treatments and during different treatment periods in a model field experiment.

The alpha diversity of microbial communities changed dynamically under different treatments and cultivation times (Figure 3). With the extension of the cultivation time, the Chao1 index of the bacterial community generally showed an upward trend. On days 0, 21, and 90, compared to the CCS and FUM treatments, the FUM + MIC treatment exhibited the highest bacterial richness and diversity, while the bacterial diversity in the FUM treatment decreased at day 90. The Chao1 and Shannon indices for the fungal community typically exhibited an upward trend as the duration of cultivation increased. On days 0, 21, and 90, compared to the CCS and FUM treatments, the FUM + MIC treatment showed the highest fungal richness and diversity. In terms of change rates, both the FUM and FUM + MIC treatments had faster rates of increase in microbial community richness and diversity (steeper slopes), with FUM + MIC being more favorable for maintaining microbial community richness and diversity throughout the entire cultivation period.

### 3.4. Microbial Community Structure Dynamics

**Figure 4:**
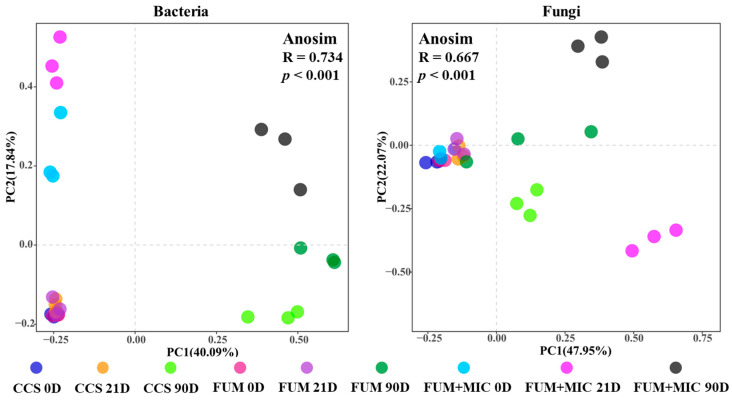
Changes in beta diversity of soil microbial communities during different cultivation periods under various treatments in a model field experiment.

Principal component analysis (PCA) results indicated significant differentiation in microbial community structure between treatments throughout the entire cultivation period (Figure 4). For the bacterial community, on day 90, the community structure of samples from all treatments was significantly separated along the PC1 axis from their corresponding samples on days 0 and 21 (*p* < 0.001). Similarly, for the fungal community, the samples on day 90 also showed a significant separation along the PC1 axis from those on days 0 and 21 (*p* < 0.001). Notably, the FUM + MIC treatment exhibited a significant separation from the day 0 community structure as early as day 21.

### 3.5. Microbial Community Composition Characteristics

**Figure 5:**
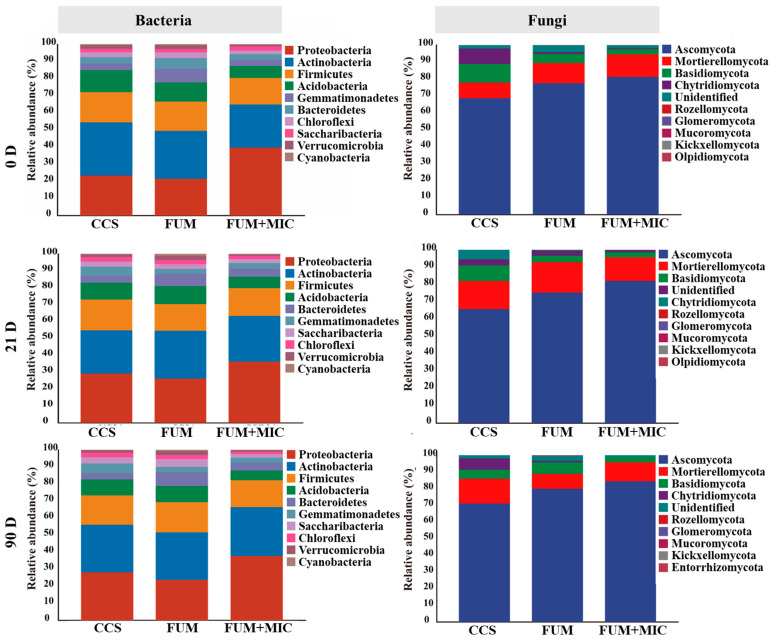
Soil microbial community composition across cultivation times under various treatments in a model field experiment.

The microbial community was different at the community composition level at the phylum level based on different treatments and cultivation durations (Figure 5). Amidst the bacterial community, the dominant phyla Proteobacteria, Actinobacteria, Firmicutes, and Acidobacteria were found to consistently dominate all treatments and sampling times within a total relative abundance reaching up to 70%. The highest relative abundance of Actinobacteria of 27.75% was observed in the CCS treatment compared to the other treatments, while the relative abundance of Proteobacteria was maximum in the FUM + MIC treatment at 37.62%. Moreover, the total relative abundance of the dominant bacterial phyla detected in the FUM + MIC treatment was higher than that in the CCS and FUM treatment.

The dominant phyla found in the fungal community include Ascomycota, Mortierellomycota and Basidiomycota, with a total relative abundance of 60% to 90%. Specifically, compared to other treatments, the relative abundance of Basidiomycota in the CCS treatment was highest at 8.51%, while in the FUM treatment, Mortierellomycota had the highest relative abundance at 13.44%, and in the FUM + MIC treatment, Ascomycota dominated with the highest relative abundance of 82.56%. The FUM + MIC treatment consistently maintained the highest total relative abundance of dominant fungal phyla across all time points, followed by the FUM treatment, with the CCS treatment showing the lowest abundance. In addition, the inoculated microbial communities were characterized. The isolates that were obtained, once identified, are 2946 species belonging to 38 phyla. Detailed information can be found in Table S2.

### 3.6. Microbial Community Co-Occurrence Network Structure and Keystone Species

**Figure 6:**
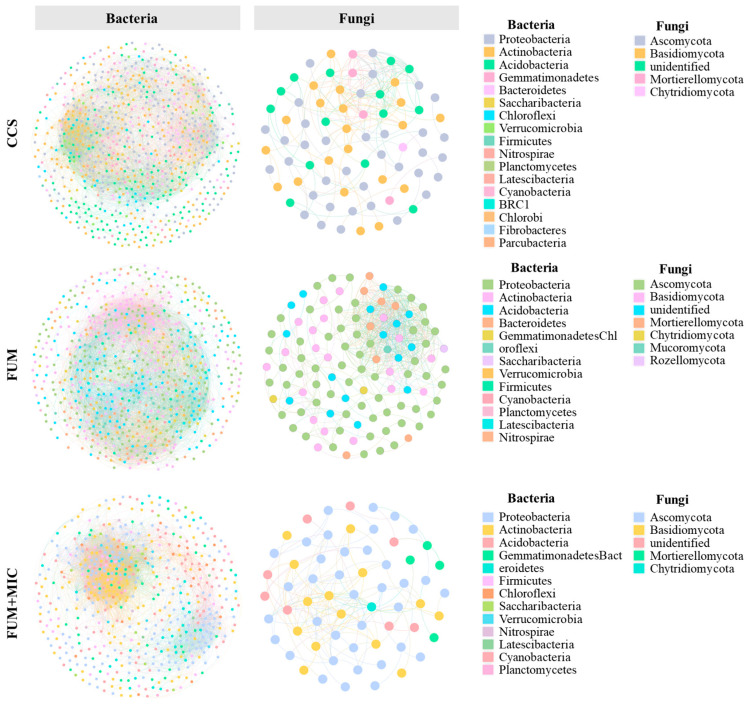
Soil microbial community co-occurrence network structure at different cultivation times under various treatments in a model field experiment.

The structure of the co-occurrence network for soil microbial communities showed notable variations depending on the treatments applied (Table 1 and Figure 6). In general, the network of bacterial communities was more intricate than that of fungal communities, featuring a greater number of nodes and linking edges. In the bacterial network, the dominant bacterial phyla involved in network formation across treatments were Proteobacteria, Actinobacteria, and Acidobacteria. Apart from the dominant phyla, certain bacterial taxa differed significantly between the treatments. Compared to CCS and FUM treatments, the FUM + MIC treatment exhibited the greatest average density and the smallest network diameter. Analysis of the subnetwork structure at different times of cultivation clearly differs in the treatments.

The Ascomycota and Basidiomycota phyla were mainly involved in network formation in the fungal network treatments. The arrangement of fungal phyla associated with network formation in the CCS and FUM + MIC treatments exhibited a notable distinction compared to that observed in the FUM treatment. The fungal network in the FUM treatment had the highest average density and the shortest average path length, whereas the FUM + MIC treatment had the smallest network diameter. Similarly, significant structural differences were observed in subnetworks at different cultivation times (Table S3).

Keystone species were identified through Zi–Pi analysis, revealing that the CCS treatment had the highest number of keystone species (23 species), but they were distributed across fewer phyla (seven phyla). In contrast, the FUM and FUM + MIC treatments had fewer keystone species, but these species were distributed across more phyla, covering six and five bacterial phyla and two and three fungal phyla, respectively (Table 2, Figure S3, Table S4).

**Figure 7:**
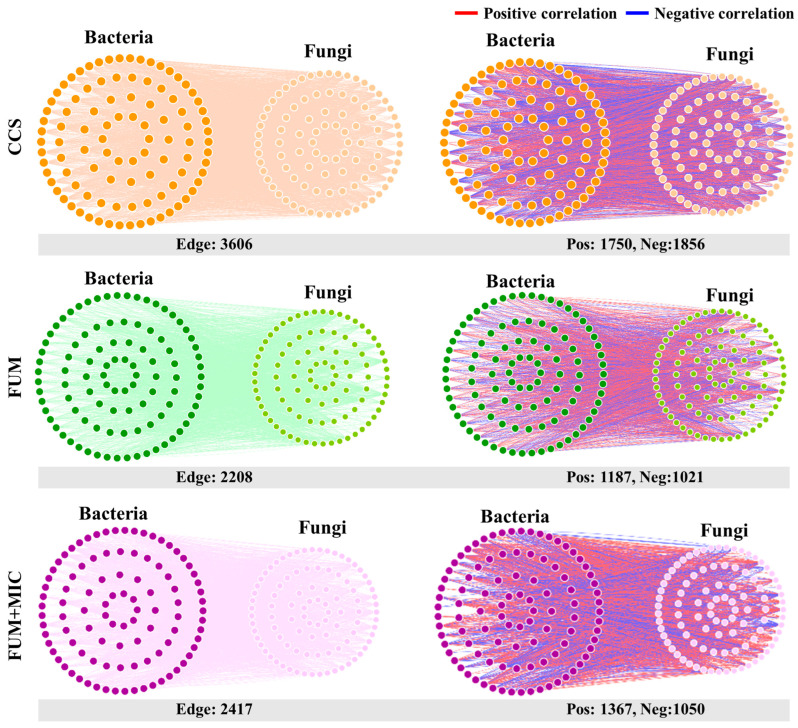
Cross-domain network of bacterial-fungal keystone species across cultivation times under various treatments in a model field experiment.

Cross-domain network analysis of keystone species (bacteria-fungi) showed that the degree of connectivity between bacteria and fungi was highest in the CCS treatment, but the proportion of positive correlations was lowest, at only 48.53% (Figure 7). The FUM treatment exhibited the lowest cross-domain connectivity. In contrast, the FUM + MIC treatment had the highest proportion of positive correlations between bacteria and fungi, at 56.56%.

### 3.7. Relationship Between Keystone Species and Soil–Plant Factors

**Figure 8:**
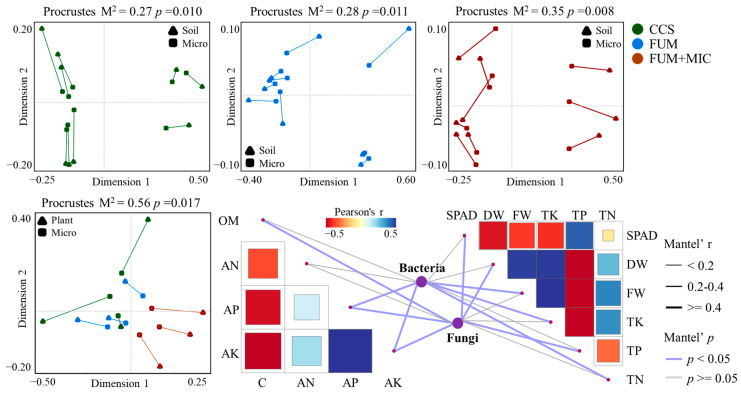
Correlation analysis among soil, plant, and microbial factors. Note: co-variation analysis between keystone species and soil (**top row**); co-variation analysis between keystone species and plants (**bottom row**, **left**); analysis of the correlations between soil and plant indicators mediated by fungi and bacteria in keystone species (**bottom row**, **right**).

The results of the procrustes analysis indicated a notable co-variation between variations in total keystone species and alterations in soil nutrient levels across various treatments and cultivation periods (*p* < 0.05) (Figure 8). Significantly, the extent of co-variation observed in the FUM + MIC treatment was markedly greater compared to the CCS and FUM treatments (*p* < 0.01). In addition, the trend of keystone changes also exhibited a significant co-variation with plant agronomic traits and nutrient accumulation characteristics (*p* < 0.05).

**Figure 9:**
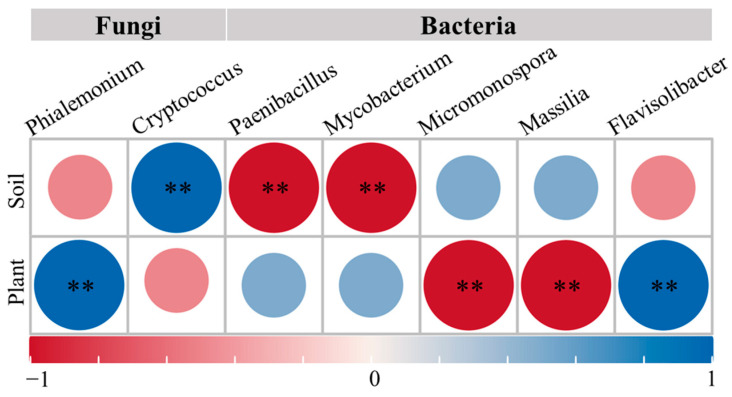
Correlation analysis between keystone genus-level species and soil and plant traits. Note: asterisks denote significant probability levels.

To further explore the potential mediating effects of keystone species on soil and plant indicators, Mantel analysis was performed to assess the correlations between bacterial and fungal keystone species and soil–plant indicators under the FUM + MIC treatment. The results showed that bacterial keystone species were strongly correlated with soil AP and AK and plant FW, TK, and TN indicators, while fungal keystone species were primarily associated with soil OM, AP, and AK and plant SPAD, DW, and TP traits. Further identification at the genus level revealed that the bacterial genera *Paenibacillus* and *Mycobacterium* and the fungal genus *Cryptococcus* were significantly correlated with soil nutrient indicators (*p* < 0.01). The bacterial genera *Micromonospora*, *Massilia*, and *Flavisolibacter* and the fungal genus *Phialemonium* were significantly correlated with plant traits (*p* < 0.01) (Figure 9).

## 4. Discussion

### 4.1. Indigenous Cultivable Microorganism Inoculation Increases Nutrient Supply and Growth of Crop in Continuous Cropping and Fumigated Soils

Soil health can be assessed using soil nutrient supply [46]. As a result of the experiment, the FUM treatment significantly reduced AP content by 6.21% on day 21 (*p* < 0.05), which indicates that while fumigation lowered harmful microorganisms it also inhibited some beneficial microbial communities’ functions. Huang and McCallister state that fumigation decreases the activity of phosphorus-solubilizing microbes like *Pseudomonas* and *Bacillus* as well as the bioavailability of phosphorus nutrients in soil [47,48]. With the extension of the cultivation time, the OM content of all three treatments tended to decline. Nonetheless, compared with continuous cropping soil, the FUM + MIC treatment significantly increased OM content by 4.66% on day 90 (*p* < 0.05). It can maintain the soil OM content, which will help to sustain soil through the inoculation of indigenous cultivable microorganisms. The reason behind this is that microbes not only assist with the breakdown and conversion of OM, thus allowing the accumulation of organic carbon in soil to be stabilized [49], but they also likely contribute to the accumulation of residues contributing to the carbon content of OM, which furthers soil health [50].

Better soil health was also evident in the plant’s phenotypes. The DW content of the plants increased significantly by 19.45% for the FUM + MIC treatment as compared to the CCS treatment. The TP content of plants under the FUM + MIC treatment was significantly higher than that under either CCS or FUM by 59.44% and 61.25%, respectively. The revitalization of the microbial community not only promoted phosphorus uptake in crops but also improved their ability to accumulate dry matter. Microbial inoculation effectively improved the mentioned crop growth traits, as found by Abou Jaoudé [51]. Soil health, fertility, and crop yield were significantly improved with the inoculation of indigenous cultivable microorganisms.

### 4.2. Enhanced Microbial Diversity and Community Structure Reconstruction Promote Ecosystem Recovery After Inoculation

Alpha diversity quantifies the richness and diversity of communities and also evaluates the efficiency of soil ecological restoration. It is an important parameter for microbial community assessment [52]. In this study, the Chao1 index of both bacterial and fungal communities increased with time. The bacterial and fungal richness on days 0, 21 and 90 of the FUM + MIC treatment were significantly higher than those under CCS and FUM treatment. This indicated that the FUM + MIC treatment could help microbes rejuvenate at a faster and more stable rate. Moreover, the FUM + MIC treatment had a higher Shannon index, indicating its ability to improve microbial diversity. Microbial communities that have high diversity can enhance the soil’s self-repairing capacity significantly through resource competition and mutualistic symbiosis. This, in turn, reduces the chances of pathogen invasion and enhances the antagonistic activity of the microbial community against pathogens [53]. Compared to the Chao1 index, the post-restoration difference of the Shannon index was less, which indicates that diversity was restored, but there may not have been a significant introduction of foreign species, and thus the ecological risks were lowered.

The FUM + MIC treatment improved bacterial community and fungal diversity on day 90 as compared to the FUM treatment. Hence, the result suggests that the inoculation of indigenous cultivable microorganisms can restore the richness and diversity of beneficial microbes rapidly, alleviating the negative impact of continuous cropping and fumigation treatments.

PCA analysis revealed significant differences in the structure of bacterial and fungal communities throughout the cultivation period. On day 90, the community structure results of the FUM + MIC treatment exhibited a significantly different structure from CCS and FUM (*p* < 0.001). It is important to note that on day 21, the fungal community under FUM + MIC was already significantly separated from day 0, signifying that indigenous cultivable microorganisms can effectively reconstruct soil microbial community structure. According to the findings of this research, soil inoculation using indigenous cultivable microorganisms may optimize the microbial composition. Significantly, this has been recorded in the dominant phyla of both bacteria and fungi. This vaccination raises their total flourishing in life. Soil nutrient cycling will also directly affect its ecological stability.

The main bacterial community phyla were Proteobacteria, followed by Actinobacteria and Firmicutes and Acidobacteria. The relative abundance of Proteobacteria was highest under the treatment of FUM + MIC at 37.62% since it is important for OC degradation [54]. Research by Ahlawat showed that the increase in the population of Proteobacteria heightens the availability of carbon sources in the soil and increases nutrient uptake by crops [55]. Mycological communities are mainly composed of Basidiomycota, Mortierellomycota, and Ascomycota. The Ascomycota relative abundance for FUM + MIC was greater at all sampling times compared with the CCS and FUM treatments. A typical group of microorganisms that can degrade organic matter is Ascomycota. Its abundant presence significantly enhances soil organic carbon transformation, which provides a more stable and abundant organic carbon source to crops [56].

Overall, the rapid community rejuvenation, soil microbial community structure reconstruction and more dominant phylum number promoted by inoculating indigenous cultivable microorganisms can provide a better growth environment for crops.

### 4.3. Inoculation Optimizes Cross-Domain Microbial Interactions and Keystone-Species-Mediated Synergistic Ecological Function Recovery

The FUM + MIC treatment significantly improved soil microbial network structure stability and optimized cross-domain synergistic interactions between bacteria and fungi, according to this study. A more stable network structure can better resist the disturbance of external ecological stress, which helps to keep the internal ecological cycles of the soil functioning and accelerate the efficiency of soil nutrient transformation and crop nutrient absorption [57,58]. The proportion of positive correlation between bacteria and fungi was 56.56% in the FUM + MIC treatment, which was higher than that in the CCS and FUM treatments. The shift suggests that the inoculation of indigenous cultivable microorganisms also improved the synergism between bacteria and fungi, helping in soil ecological function restoration. Furthermore, stronger community stability and positive synergistic interactions have been reported to bolster microbial antagonism toward pathogens [59].

The Zi–Pi assessment revealed that despite having the minimum amount of keystone species, the FUM + MIC treatment had its keystone species dispersed across more phyla, indicating these keystones’ roles may be more important after inoculation. Their interactions with other microorganisms facilitated diversification and functional complementarity in communities [60]. The complementary analysis through procrustes analysis indicates that the correlation between soil nutrients and plant growth indicators (FW, WD, and SPAD and TN, TP, and TK contents) with keystone species was higher in the FUM + MIC treatment. These keystone species played a crucial role in transforming the nutrients carbon, phosphorus, and potassium [61].

Moreover, the researchers also found that bacteria are more likely to affect soil available nutrients and plant nutrient accumulation, while fungal keystone species mostly affect carbon accumulation and plant agronomic traits. This happens due to that bacteria decompose OM rapidly to release mineral nutrients, which directly enhance soil available nutrients and promote plant nutrient uptake [62]. On the other hand, fungi, through their hyphal networks and mycorrhizal associations, enhance water and mineral uptake by plants and also increase soil organic carbon by decomposing complex organic materials that affect plant carbon use and growth attributes [63]. Specifically, *Paenibacillus* and *Mycobacterium* from the bacterial and *Cryptococcus* from the fungal community dramatically impact compositions of soil nutrient transformations, as reported also by Miao and Grady [64,65]. In addition, various other microorganisms such as *Micromonospora*, *Massilia*, *Flavisolibacter*, *Phialemonium* and many more are key for plant growth promotion in corn, alfalfa, tomato, etc. [66,67].

In conclusion, the inoculation of indigenous cultivable microorganisms, by optimizing cross-domain interactions between bacteria and fungi and regulating keystone species, can enhance the transformation of carbon, nitrogen and phosphorus in the soil for healthier crop growth. In addition, future studies will systematically evaluate the direct effects of indigenous cultivable microorganism inoculation on soil pathogens in continuous cropping systems, aiming to determine whether it can directly suppress disease, improve soil health, and sustain crop growth. Future research will focus on monitoring changes in the living microbial communities following inoculation, as well as applying macro-cultivation techniques to extract a greater diversity of native, non-cultivable microorganisms from soil. This approach will improve the representativeness and diversity of microbial communities, offering a deeper insight into soil microorganisms and their ecological impact on soil health.

## 5. Conclusions

Inoculation with indigenous cultivable microorganisms can rapidly restore soil microbial communities in continuously cropped and fumigated soils to improve soil functions and plant growth, our study shows. After the inoculation, the bacterial and fungal community richness and diversity of the soil increased significantly along with a shift in community structure. Moreover, network stability of the soil was greatly improved after the inoculation. The positive interactions between bacteria and fungi from different species were greatly improved. Soil AP and AK content were modulated by bacterial fungal keystone species like *Paenibacillus* and *Mycobacterium*, while *Micromonospora*, *Massilia* and *Flavisolibacter* regulated plant FW, TK and TN content. Moreover, the fungal keystone species *Cryptoccocus* affected OM, AP, and AK, while *Phialemonium* influenced plant SPAD, DW and TP. Ultimately, inoculation sustained the relative equilibrium of OM and dramatically enhanced the accumulation of DW and TP in plants. According to this finding, the efficacy and sustainability of indigenous cultivable microorganism inoculation are ecologically viable. It offers a new opportunity for the restoration of soil problems affected by continuous cropping.

## Figures and Tables

**Table 1:** Topological features of soil microbial community networks under various treatments in a model field experiment.

Treatments		Nodes	Edges	Average_ Density	Average_ Path.length	Network_ Diameter
Bacteria	CCS	759	9461	24.930	3.429	17.285
	FUM	682	10,602	31.091	3.186	17.033
	FUM + MIC	546	10,291	37.696	3.437	13.301
Fungi	CCS	76	188	4.947	4.209	12.770
	FUM	112	573	10.232	2.338	8.082
	FUM + MIC	63	146	4.635	2.368	6.320

**Table 2:** Statistical summary of total keystone species across cultivation times under various treatments in a model field experiment.

	Keystone	OTU (Number)	Phylum (Number)	Class (Number)	Order (Number)	Family (Number)	Genus (Number)
Bacteria	CCS	21	6	13	14	15	11
	FUM	15	6	6	9	9	7
	FUM + MIC	9	5	7	6	3	3
Fungi	CCS	2	1	2	2	2	2
	FUM	3	2	2	1	0	0
	FUM + MIC	7	3	4	4	6	5

## Data Availability

The sequence files were submitted to the NCBI Sequence Read Archive repository (http://www.ncbi.nlm.nih.gov/sra, accessed on 26 March 2026) and are accessible with the Ascension Numbers Bio Project PRJNA978996, PRJNA1417082, and PRJNA1417100.

## Supplementary Materials

The following supporting information can be downloaded at: https://www.mdpi.com/article/10.3390/microorganisms14040784/s1, Figure S1: Method of soil sample collection; Figure S2: Research area location map; Figure S3: Total soil keystone selection at different cultivation times under different treatments; Table S1: Composition of the culture medium; Table S2: Identification results of inoculated indigenous microorganisms; Table S3: Topological features of soil microbial community networks across cultivation times under various treatments in a model field experiment; Table S4: Detailed information of keystone species.
