# Supplementary material for: The Effect of Indigenous Cultivable Microorganism Inoculation on Soil Microecology During Restoration of Obstructed Soils
Source: Microorganisms. 2026 Mar 30;14(4):784. doi: 10.3390/microorganisms14040784 (PMC13118302; doi:10.3390/microorganisms14040784)
Supplement: Supplementary file 1 [file microorganisms-14-00784-s001.zip › Supplementary Information.pdf]

The Effect of Indigenous Cultivable Microorganism Inoculation on Soil Microecology During Restoration of Obstructed Soils

Qunfei Ma, Bing Zhang * and Juntao Cui *

College of Resources and Environment, Jilin Agricultural University, Changchun 130118, China;
*   Correspondence: zb18686446806@126.com (B.Z.); juntaoc@jlau.edu.cn (J.C.)

Supplementary tables

Table S1 Composition of the culture medium.

Microorganisms	Culture medium
**Bacteria**	Beef Extract Peptone Agar: Beef extract 3 g, peptone 10 g, NaCl 5 g, agar 20 g, water 1000 ml, pH 7.0-7.2, 1.05 kg/cm², 121°C for 20 min.
**Fungi**	Czapek's Agar: $NaNO_3$ 2 g, K_2HPO_4 1 g, KCl 0.5 g, $MgSO_4$ 0.5 g, $FeSO_4$ 0.01 g, sucrose 30 g, agar 20 g, water 1000 ml, natural pH, streptomycin 1%, 1.05 kg/cm², 121°C for 20 min.
**Actinomycetes**	modified Gao I agar: Soluble starch 20 g, KNO_3 1 g, NaCl 0.5 g, K_2HPO_4 0.5 g, $MgSO_4$ 0.5 g, $FeSO_4$ 0.01 g, agar 20 g, water 1000 ml, pH 7.2-7.4, potassium dichromate 0.5%, 1.05 kg/cm², 121°C for 20 min.

Table S2 Identification results of inoculated indigenous microorganisms.
(see Table S2.xlsx)

Table S3 Topological features of soil microbial community networks across cultivation times under various treatments in a model field experiment.

	Treatments	Time	Nodes	Edges	Average_ density	Average_ path.length	Network_ diameter
Bacteria	CCS	0 D	716	8211	0.032	3.469	16.069
		21 D	709	8227	0.033	3.462	17.290
		90 D	663	6750	0.031	3.554	17.544
	FUM	0 D	655	9883	0.046	3.266	18.224
		21 D	652	9839	0.046	3.304	17.669
		90 D	497	4937	0.040	3.261	14.412
	FUM+MIC	0 D	526	9939	0.072	3.450	13.318
		21 D	501	9471	0.076	3.385	12.992
		90 D	470	6109	0.055	3.599	12.500
Fungi	CCS	0 D	60	102	0.054	3.669	10.052
		21 D	67	128	0.057	3.721	10.402
		90 D	60	148	0.083	3.355	10.015
	FUM	0 D	107	527	0.093	2.523	8.661
		21 D	106	554	0.100	2.287	8.661
		90 D	84	342	0.095	2.351	8.287
	FUM+MIC	0 D	58	116	0.071	2.325	6.464
		21 D	55	119	0.080	2.286	6.401
		90 D	47	76	0.071	2.242	5.539

Table S4 Detailed information of keystone species.
(Table S4.xlsx)

Supplementary figure

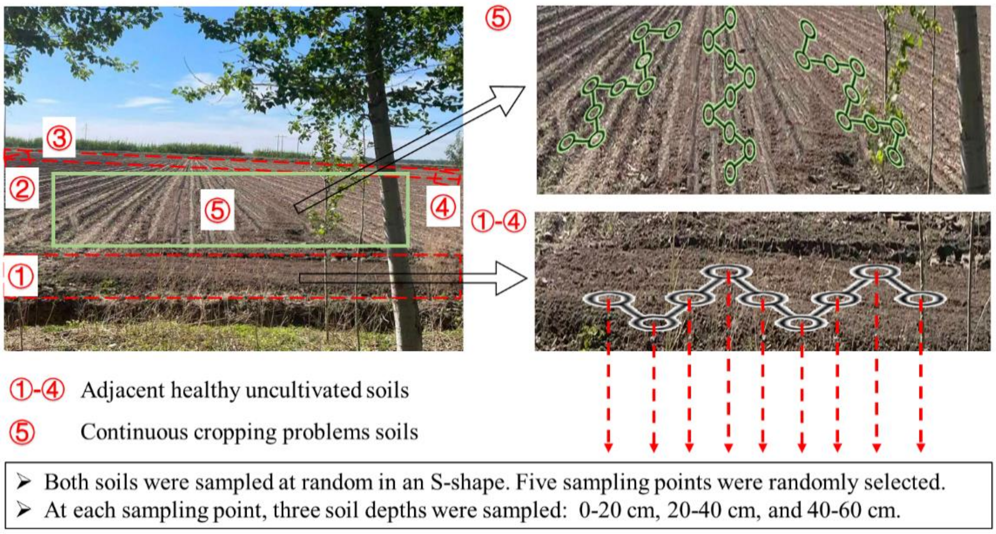

Figure S1. Method of soil sample collection

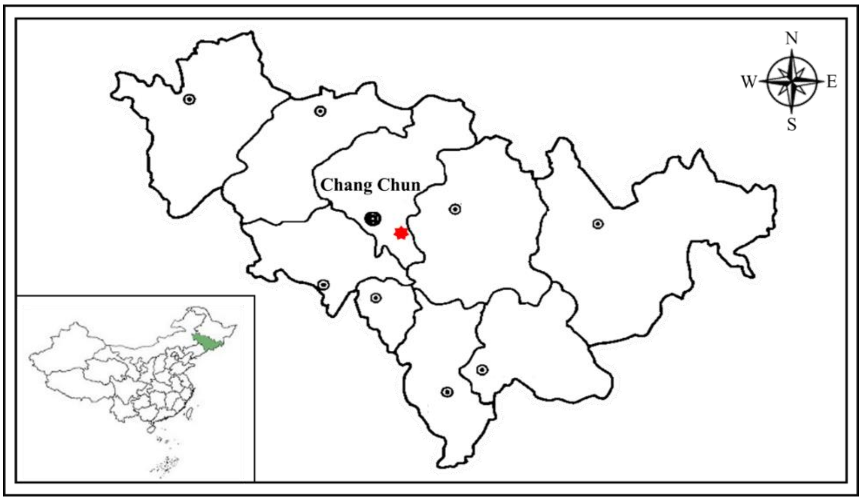

Figure S2. Research area location map

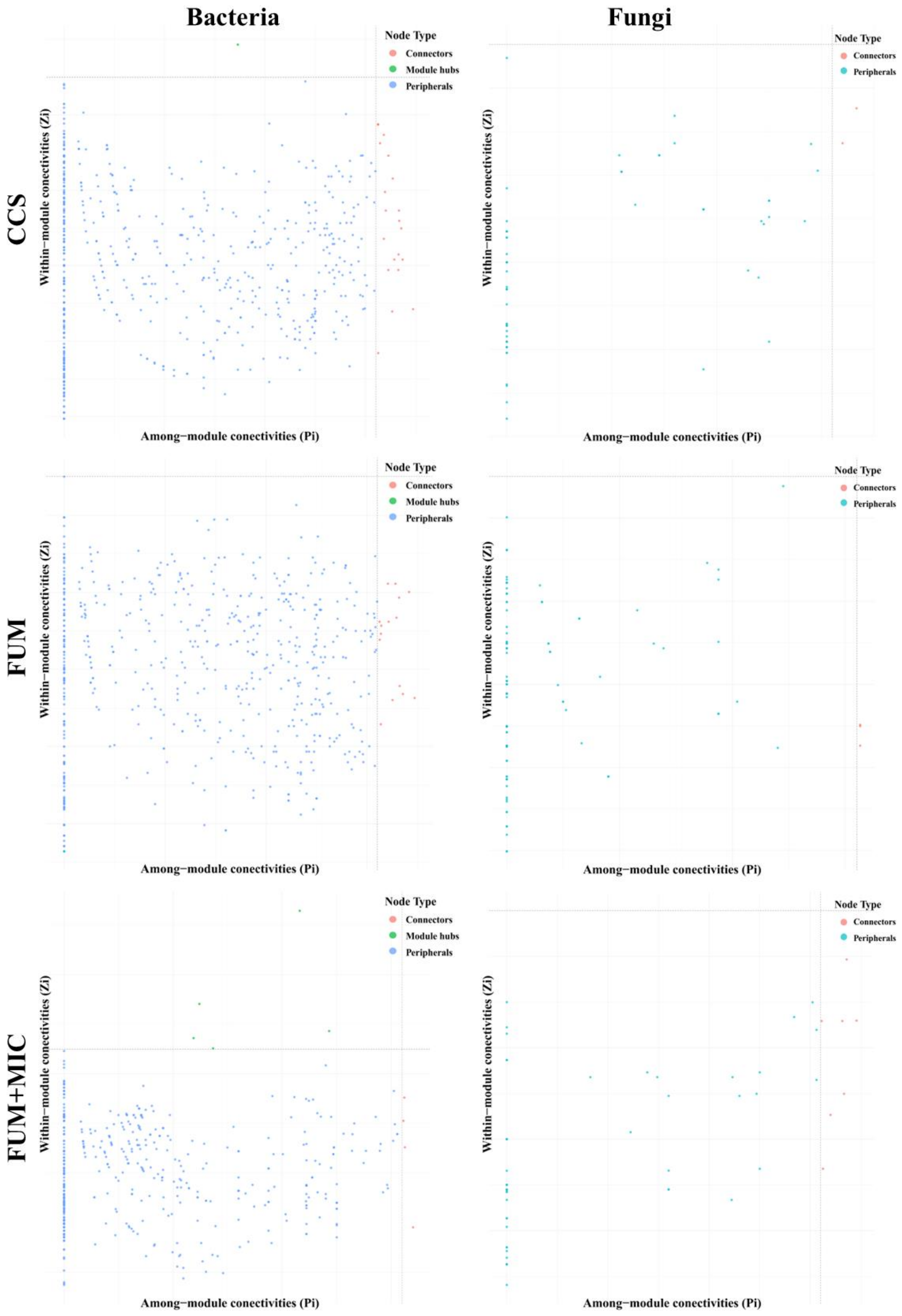

Figure S3. Total soil keystone selection at different cultivation times under different treatments
